# NAADP-Dependent Ca^2+^ Signaling Controls Melanoma Progression, Metastatic Dissemination and Neoangiogenesis

**DOI:** 10.1038/srep18925

**Published:** 2016-01-06

**Authors:** Annarita Favia, Irene Pafumi, Marianna Desideri, Fabrizio Padula, Camilla Montesano, Daniela Passeri, Carmine Nicoletti, Augusto Orlandi, Donatella Del Bufalo, Manuel Sergi, Elio Ziparo, Fioretta Palombi, Antonio Filippini

**Affiliations:** 1 Department of Anatomy, Histology, Forensic Medicine and Orthopaedics, Unit of Histology and Medical Embryology,SAPIENZA University of Rome, 16 Via A. Scarpa, 00161 Rome, Italy; 2 Experimental Chemotherapy Laboratory, Regina Elena National Cancer Institute, 53 Via E. Chianesi, 00144, Rome, Italy; 3 Department of Chemistry, SAPIENZA University of Rome, 5 Piazzale Aldo Moro, 00185 Rome, Italy; 4 Department of Biomedicine and Prevention, Tor Vergata University of Rome, Via di Tor Vegata, 00173 Rome, Italy; 5 Faculty of Biosciences and Technologies for Food, Agriculture and Environment, University of Teramo, 1 Via R. Balzarini, 64023 Teramo, Italy

## Abstract

A novel transduction pathway for the powerful angiogenic factor VEGF has been recently shown in endothelial cells to operate through NAADP-controlled intracellular release of Ca^2+^. In the present report the possible involvement of NAADP-controlled Ca^2+^ signaling in tumor vascularization, growth and metastatic dissemination was investigated in a murine model of VEGF-secreting melanoma. Mice implanted with B16 melanoma cells were treated with NAADP inhibitor Ned-19 every second day for 4 weeks and tumor growth, vascularization and metastatization were evaluated. Control specimens developed well vascularized tumors and lung metastases, whereas in Ned-19-treated mice tumor growth and vascularization as well as lung metastases were strongly inhibited. *In vitro* experiments showed that Ned-19 treatment controls the growth of B16 cells *in vitro*, their migratory ability, adhesive properties and VEGFR2 expression, indicating NAADP involvement in intercellular autocrine signaling. To this regard, Ca^2+^ imaging experiments showed that the response of B16 cells to VEGF stimulation is NAADP-dependent. The whole of these observations indicate that NAADP-controlled Ca^2+^ signaling can be relevant not only for neoangiogenesis but also for direct control of tumor cells.

Vascular endothelial growth factor (VEGF) and its receptor VEGFR2 play a pivotal role in stimulating angiogenesis, including vascularization of solid tumors^1^. Angiogenesis is an important step in the outgrowth of tumors^2^ controlling their dissemination, progression and metastasis. Therapeutic targeting of angiogenesis is therefore an important challenge, still in need of basic research. In particular, no clinical tool is at present successful in inhibiting the long-term response to VEGF, the most important angiogenic factor overexpressed by the vast majority of human cancers^3^. Owing to the central role of VEGF, the first angiogenic inhibitors designed for clinical use were targeted against VEGF and its tyrosine kinase receptor^4^. VEGF neutralizing antibodies and some multi-target tyrosine kinase inhibitors have been approved for clinical use, and many more are being evaluated in clinical trials^5^. Although these therapies provide improvement in progression-free or overall survival, they are nonetheless met with drug tolerance and side effects, attesting to the complexity of VEGF signaling and tumor angiogenic cascade. Anti-VEGF treatments can induce adverse effects, possibly due to the blockade of the entire complex of VEGF signaling pathways resulting in ‘off-target’ effects^6^. These findings suggest that the challenge represented by therapeutic targeting of angiogenesis in advanced cancer is an interesting aim to further pursue through better characterization of the intracellular pathways involved in the response to VEGF.

Melanoma is one of the most aggressive and highly metastatic cancers. It is known that VEGF levels are associated with melanoma progression^10^,^11^ and are used as prognostic indicators^12^, but the molecular mechanisms regulating the interaction between tumor and endothelial cells (ECs) and their role in cancer cell extravasation and metastasis are still unclear. Several studies have shown the vascular system to be pivotal for metastasis in melanoma. Consequently, the effect of different antiangiogenic therapies has been and is being investigated in preclinical and clinical trials focusing on inhibition of either VEGF signaling or different angiogenic factors^13^. However, overall survival rates have not significantly improved in clinical trials with antiangiogenic agents due to the resistance to anti-VEGF therapy. Therefore the therapy of malignant melanoma remains challenging.

Both tumor growth and metastatic spreading depend on the formation of new blood vessels, to promote which tumor cells secrete angiogenic growth factors that act on the neighbouring ECs, inducing proliferation and migration. These processes are highly controlled by the ubiquitous second messenger Ca^2+^, recognized as a relevant factor in the growth of tumors and metastatic behaviour of cancer cells and focus of a novel area of research in oncology^14^,^15^. Cancer cells present alterations in Ca^2+^ fluxes across the plasma membrane that reflect changes in the expression, localization and/or function of different types of Ca^2+^ channels. In particular, the expression of different members of the transient receptor potential (TRP) family has been shown to be altered in cancer cells. In melanoma different transient receptor potential melastatins (TRPM) are expressed; several studies point to TRPM1 as responsible for tumor suppression, and both metastatic progression and aggressiveness of melanoma are correlated to the loss of TRPM1 expression^16^,^17^. Moreover, in many tumors other than melanoma, depletion of Ca^2+^ from the ER may drive tumor growth by inducing Ca^2+^ influx through the plasma membrane, mediated by the store operated calcium entry (SOCE) canonical components. Endoplasmic Ca^2+^ depletion sensor (STIM1) translocates to the plasma membrane and activates Ca^2+^ influx channel (ORAI1). These channels are highly expressed in human melanoma and in melanoma cell lines^18^. Voltage gated calcium channels (VGCCs) are also involved in melanoma progression by generating oscillatory Ca^2+^ waves that favour cell cycle progression^19^. These data show that regulation of intracellular Ca^2+^ has an important impact on melanoma growth and metastasis, but little is known about the involvement of intracellular calcium ([Ca^2+^]_i_) release in melanoma. It is known that calcium release from endoplasmic reticulum (ER) via PLC/IP_3_ pathway is involved in melanoma cell migration, invasion and metastasis^20^,^21^. In addition, Vartarian *et al*. highlighted the importance of both extracellular and intracellular Ca^2+^ concentration in integrin distribution and cytoskeleton remodelling in melanoma^22^. Increases in [Ca^2+^]_i_ are regulated by different Ca^2+^-mobilizing messengers: besides IP_3_ and cyclic adenosine diphosphoribose (cADPR), which mobilize Ca^2+^ from ER stores, nicotinic acid adenine dinucleotide phosphate (NAADP) has been more recently identified as capable of triggering Ca^2+^ release from acidic organelles, such as lysosomes and endosomes^23^,^24^ and appears to be a pivotal trigger for many Ca^2+^ signaling events^25^. NAADP likely targets two-pore channels (TPCs)^26^,^27^ which are distinct from IP_3_ receptors (IP_3_Rs) and ryanodine receptors (RyRs), and the resulting localized NAADP-evoked Ca^2+^ signals may in some cases be globalized via IP_3_Rs and RyRs through Ca^2+^-induced Ca^2+^ release^28^,^29^,^30^. A powerful tool in NAADP signaling characterization is represented by the selective membrane-permeant non competitive antagonist Ned-19, which blocks NAADP-mediated Ca^2+^ signaling^31^,^32^,^33^.

We have recently demonstrated in ECs the direct relationship between NAADP-mediated Ca^2+^ release and the specific signaling mechanism controlling angiogenesis^33^: the detection of a master NAADP/TPC2/Ca^2+^ machinery controlling VEGF-induced angiogenesis could contribute to the identification of new targets for therapeutic strategies. On this basis, and given the paracrine dialogue between endothelium and tumor, we have investigated the direct and indirect involvement of NAADP-mediated Ca^2+^ signaling on melanoma proliferation, invasivity and angiogenesis. Our results indicate that specific targeting of NAADP –dependent Ca^2+^ release *in vitro* and *in vivo* results in the twofold benefit of exerting both antitumor and antiangiogenic effects.

## Results

### *In vivo* toxicology and pharmacokinetic parameters of Ned-19

**Figure 1:**
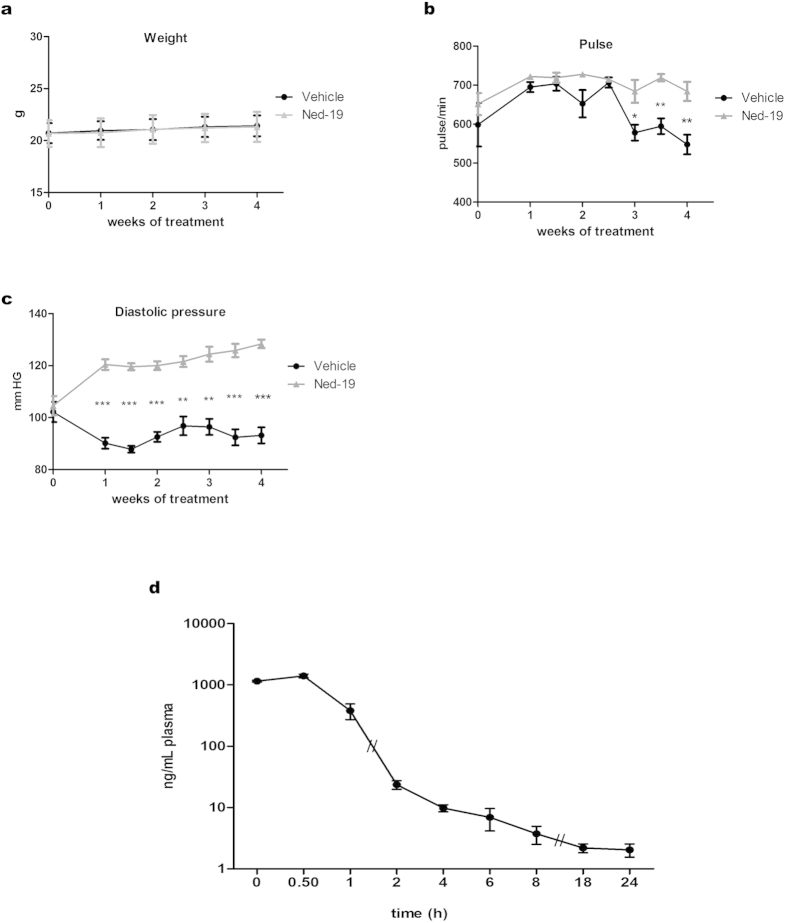
Vital parameters and analysis of Ned-19 pharmacokinetics in C57BL/6 mice. (**a–c**) Measuments of mice weight, pulse rate and diastolic pressure after i.p. injection of 5 mg/Kg Ned-19 every 48 h over four weeks. Each point is the mean of five independent measurements ± s.e.m. (**d**) Time course of Ned-19 concentrations in mouse plasma following i.p. administration (5 mg/Kg Ned-19). Each point is the mean of four replicates ± s.e.m. *P < 0.05; **P <  0.01; ***P < 0.0001.

In order to test the safety of Ned-19 treatment *in vivo*, we performed toxicological studies on adult male C57BL/6 mice intraperitoneally injected with different concentrations (5, 20 mg/Kg) of Ned-19. Diastolic pressure, pulse rate and weight were monitored once a week. When intraperitoneally injected in mice over a period of 4 weeks, 5 mg/Kg Ned-19 did not substantially affect either pulse rate or weight, while diastolic pressure, though variable, remained within physiological values (Fig. 1a–c). Moreover, this schedule of Ned-19 administration did not produce any adverse effects as monitored by postural and behavioral changes. The *in vivo* biopharmaceutical profile of Ned-19 was studied by measuring pharmacokinetic parameters in plasma samples, obtained at fixed times after one intraperitoneal (i.p) injection of 5 mg/Kg Ned-19. Results showed that Ned-19 was very rapidly absorbed reaching maximum plasma concentrations 30 min after injection (Fig. 1d).

### Ned-19-mediated inhibition of tumor growth and vascularization

**Figure 2:**
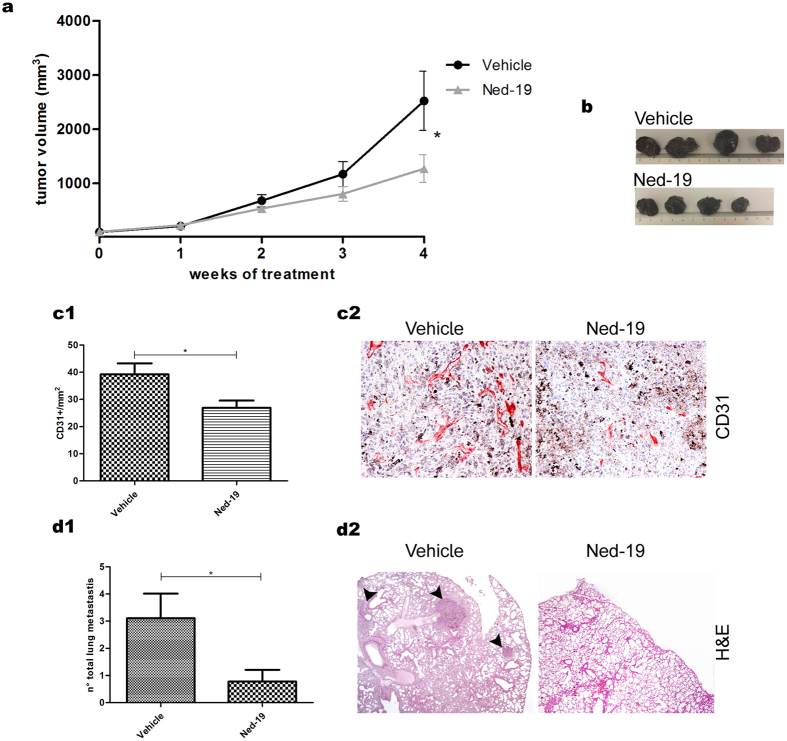
Ned-19 inhibits the growth, vascularization and metastatic potential of B16 tumors. (**a,b**) B16 cells were subcutaneously inoculated in C57BL/6 mice and treated as indicated in Material and Methods. Analysis of tumour size increase (mm^3^) in mice injected i.p. with Ned-19 or DMSO (vehicle). (**a**) Graph of a representative experiment (mean ± s.e.m). Each group consists of minimum 6 mice. Comparable results were obtained in three independent experiments. (**b**) Representative pictures of tumours from mice treated as indicated. (**c**) Tumor vascularization in samples treated or not with Ned-19 for four weeks. ECs were recognized as CD31+. (**c1**) Vessel density. (**c2**) Representative histological sections of tumors immunostained for CD31 (red). (**d**) Lung metastastes from mice treated or not with Ned-19 for four weeks. (**d1**) Number of total metastases. (**d2**) Representative H&E stained histological sections in which metastases are indicated by arrow heads. Where applicable, values are expressed as mean ± s.e.m. from three independent experiments. *P < 0.05.

On the basis of the safety and pharmacokinetic parameters reported above, every second day administration of 5 mg/Kg Ned-19 was chosen to investigate the effect of this inhibitor on tumor growth and angiogenesis in mice inoculated with B16 cells. Our results show that, in comparison to vehicle-treated specimens, tumor growth was severely impaired by 4-week i.p. Ned-19 administration (Fig. 2a,b) in the absence of adverse health effects, suggesting the involvement of NAADP signaling in this tumor. In fact, the comparative histological analysis of tumors from treated and untreated mice showed that the density of CD31 positive tumor vessels was reduced by Ned-19 treatment (Fig. 2c). An additional tumor feature affected by Ned-19 was its metastatic behaviour. Since melanoma B16 is known to induce the formation of spontaneous metastasis in lungs, fixed lungs of Ned-19-treated and untreated mice were macroscopically and microscopically inspected for the presence of metastatic nodules. Ned-19 was found to drastically reduce the formation of total lung metastases (micro and macro- metastases) (Fig. 2d), possibly suggesting impaired melanoma cell entry into the blood flow following Ned-19-induced reduction of primary tumor vascularization. The hypothesis of a possible contribution from a direct response of melanoma cells to Ned-19 is addressed in the experiments described below.

### Evaluation of VEGF-induced Ca^2+^ release in melanoma B16 cells

VEGF is produced by a large variety of tumors, including melanoma^34^, in which it appears to play an important role in the process of tumor-induced angiogenesis^3^. Several studies in the literature have focused on the presence in melanoma cells, but not in melanocytes, of the VEGF receptors VEGFR2^36^,^37^ and, more recently, VEGFR1^38^.

**Figure 3:**
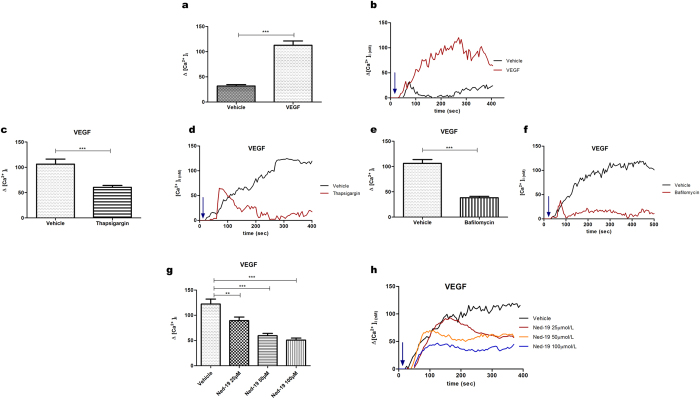
VEGF mobilizes calcium activating Ca^2+^ release from acidic stores. Live imaging in single FURA-2AM loaded cells; representative traces and maximum Ca^2+^ concentrations, shown as bar charts. Intracellular Ca^2+^ levels in B16 cells after stimulation with 100 μg/L VEGF: (**a**) bar charts, (**b**) representative traces. (**c–f**) Identification of VEGF activated intracellular Ca^2+^ stores. Ca^2+^ release in cells stimulated with 100 μg/L VEGF after treatment with either vehicle alone (control) or (**c,d**) 1 μmol/L thapsigargin for 15 min or (**e,f**) 0.5 μmol/L bafilomycin A1 for 1 h. (**g,h**) Cells were pretreated with 25, 50 or 100 μmol/L Ned-19 for 30 min, then stimulated with 100 μg/L VEGF. Arrow indicates time of agonist addition. Each data point in bar charts represents mean ± s.e.m. from three to five independent experiments, n = 35–139 cells. **P < 0.01; ***P < 0.0001.

It is known that in melanoma cell lines several biological processes are controlled by [Ca^2+^]_i_ 20,21. To characterize the possible contribution of VEGF to Ca^2+^ signaling, B16 cells were stimulated with VEGF_A165_ (known as VEGF) and [Ca^2+^]_i_ was assayed in FURA-2AM loaded single cells. Figure 3a,b show that VEGF stimulation triggers Ca^2+^ signaling in B16 melanoma cells. In order to evaluate the possible involvement of NAADP-dependent intracellular compartments in VEGF-induced Ca^2+^ mobilization we adopted a pharmacological approach using thapsigargin, which blocks ER SERCA pumps and bafilomycin A1, which inhibits pH-dependent Ca^2+^ uptake into acidic stores by inhibition of the vacuolar-type H^+^-ATPase pump^23^. VEGF-induced Ca^2+^ release was significantly impaired not only by thapsigargin (Fig. 3c,d), but even more by bafilomycin A1 (Fig. 3e,f), showing the involvement of acidic Ca^2+^ stores in the response to VEGF. The role of NAADP indicated by these experiments was then confirmed by the observation that pretreatment of B16 cells for 30 min with 25, 50 and 100 μmol/L Ned-19 concentration-dependently inhibits VEGF-induced Ca^2+^ release (Fig. 3g,h). These experiments show for the first time that NAADP controls VEGF-evoked Ca^2+^ signaling in B16 cells.

### B16 cell migration is modulated by NAADP-dependent Ca^2+^ release

**Figure 4:**
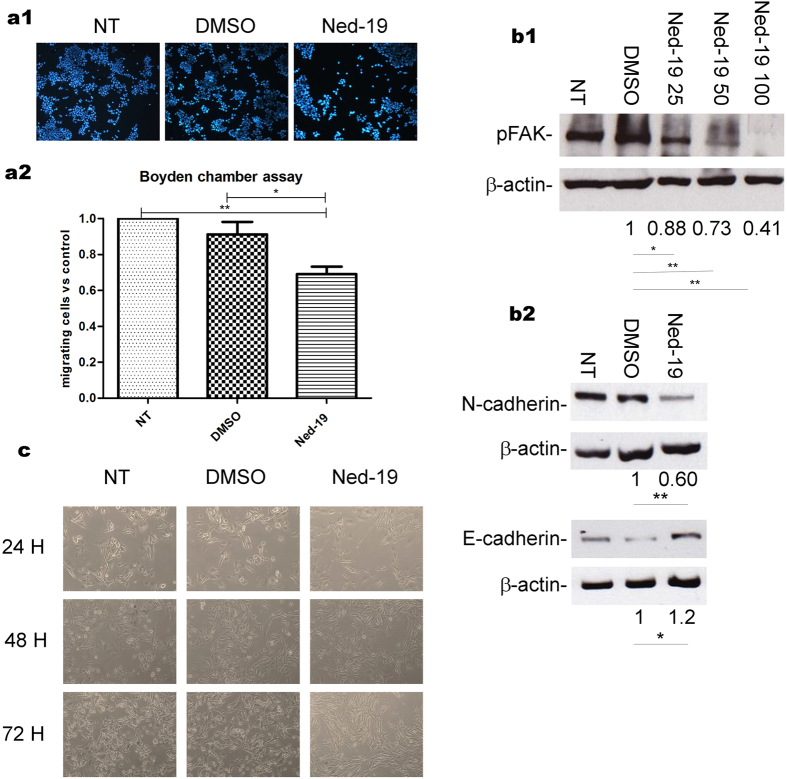
Ned-19 inhibits B16 cell migration and adhesivity. (**a**) Cells treated as indicated were allowed to migrate for 24 h across the membrane in Boyden chambers. (**a1**) Cells that migrated into the filter were Hoechst stained (**a2**) and counted in 20 fields per well. Results are expressed as migrating cells vs non-treated control (NT). (**b1,b2**) Cell lysates tested by Western blotting with a p-FAK specific antibody, anti E-cadherin, anti N-cadherin. β-actin was used as loading control. (**c**) Phase contrast images of B16 cells coltured in the indicated conditions. Data shown in (**b**,**c**) and their respective densitometric values are representative of three independent experiments. Where applicable, values are expressed as mean ± s.e.m. from three to five independent experiments. *P < 0.05; **P < 0.01.

Tumor growth and metastatic spreading depend on the formation of new blood vessels, which tumor cells stimulate by paracrine secretion of angiogenic growth factors. Both proliferative and migratory behaviour of melanoma cells are known to be highly controlled by the ubiquitous second messenger Ca^2+^. Given the observed role of NAADP in the response of B16 cells to VEGF *in vitro*, we investigated whether also the proliferative and migratory ability of these cells is controlled by this second messenger. Boyden chamber migration assays (Fig. 4a) clearly showed that melanoma cell migration is reduced by Ned-19 treatment. Cell migration and invasion of extracellular matrix involve reorganization of the actin cytoskeleton. We observed that Ned-19 significantly reduced the activation of the focal adhesion kinase (FAK) known to play a key role in regulating the dynamic changes in actin cytoskeleton reorganization (Fig. 4b1). In melanoma progression, metastasis is accompanied by down-regulation of E-cadherin and up-regulation of N-cadherin expression, which facilitate the separation of melanoma cells from adjacent E-cadherin-expressing keratinocytes and the invasion of the dermal tissue through interactions with the N-cadherin-expressing fibroblasts^39^. Treatment of melanoma cells with Ned-19 resulted in reduced expression of N-cadherin and increased expression of E-cadherin, affecting the cell migratory behaviour (Fig. 4b2). This was paralleled by cell shape changes apparent in phase contrast microscopy during 24, 48 and 72 h Ned-19 treatment (Fig. 4c). NAADP-mediated Ca^2+^ release is thus required for the migratory ability of B16 cells in their own conditioned medium.

### Ned-19 inhibits B16 melanoma cell viability, proliferation and VEGFR2 expression

**Figure 5:**
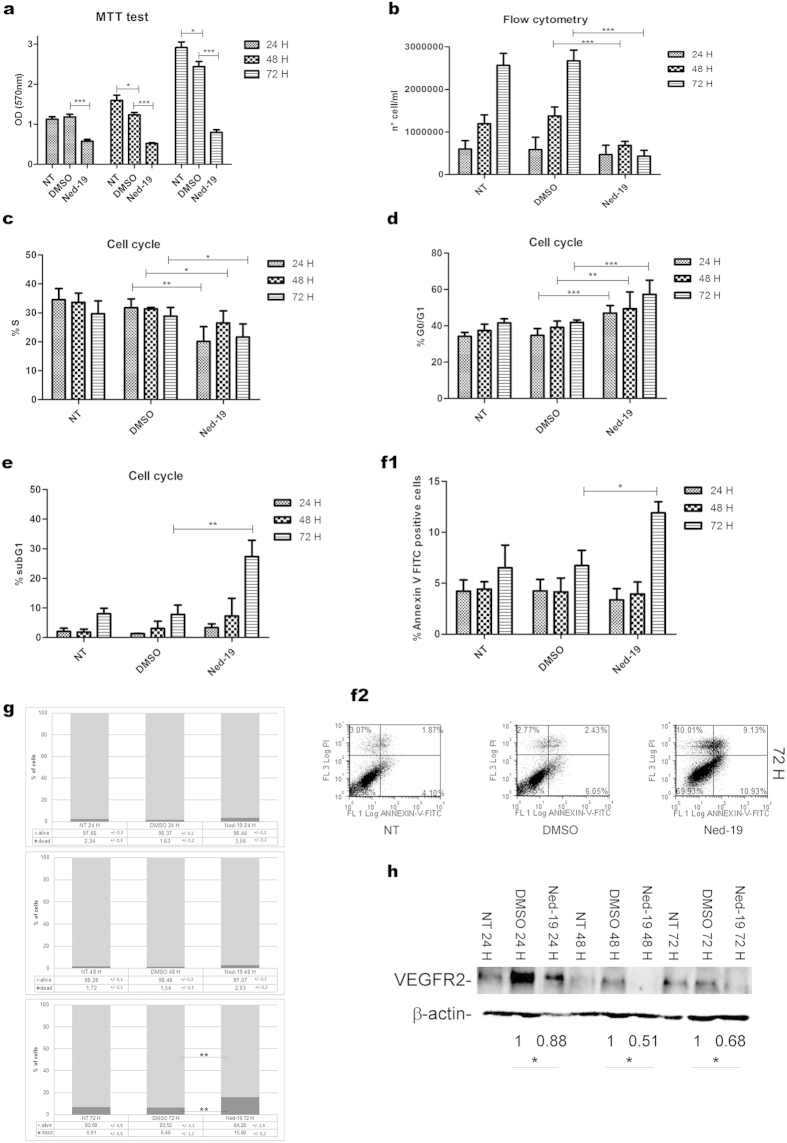
Ned-19 affects B16 cells proliferation rate, cell viability and VEGFR2 expression. Cells were treated with Ned-19 or vehicle (DMSO) as indicated for 24, 48, 72 h. (**a**) Cell viability quantified as OD (570 nm) by MTT assay. (**b**) Number of cells evaluated by flow cytometry. (**c–e**) Evaluation of % S phase, % G0/G1 phase, % subG1 population by cell cycle analysis. (**f1**) The percentage of annexin V- FITC positive cells studied by flow cytometry is shown in the histogram. (**f2**) Annexin V-FITC/ PI representative dot plot of cells treated for 72 h with Ned-19; lower right quadrant: apoptotic cells; upper right and left quadrants: necrotic cells; lower left quadrant: live cells (for quadrants setting details see Methods section). (**g**) The percentage of live and dead cells in stimulated and control conditions was tested by flow cytometry using PI. (**h**) Cell lysates tested by Western blotting with a VEGFR2 specific antibody. β-actin was used as loading control. Data shown in (**h**) and their respective densitometric values are representative of three independent experiments. Values are expressed as mean ± s.e.m. from three to five independent experiments. *P < 0.05; **P < 0.01; ***P < 0.0001.

The involvement of NAADP in B16 cell proliferation was evaluated using the NAADP antagonist Ned-19 and measuring different indicators. The number of viable cells, assessed by the thiazolyl blue tetrazolium bromide (MTT) assay for spectrophotometric determination of cell viability, was reduced from 24 h up to 72 h following Ned-19 treatment (Fig. 5a). Flow cytometry assessment showed that the cell number was markedly reduced by Ned-19 while control cells, either untreated or vehicle-treated, underwent exponential growth (Fig. 5b). The reduction in cell number resulting from Ned-19 treatment was consistent with the reduction of the S phase percentage and the increase of the G0/G1 phase percentage evaluated by cell cycle analysis (Fig. 5c,d). These results suggest that melanoma B16 cell proliferation is dependent upon NAADP-mediated Ca^2+^ release, showing that Ned-19 treated cells are arrested in S phase and undergo an increase in % subG1 population after 72 h (Fig. 5e). To get insight on the composition of the subG1 population, samples were further analysed in flow cytometry by annexin V-FITC (Fig. 5f1,f2) and propidium iodide (PI) (Fig. 5g) staining, demonstrating that the percentage of apoptotic cells, which was highest at 72 h, was less than 15%.

Having observed the effect of NAADP inhibition on B16 cell proliferation and migration we wondered whether VEGFR2 expression was also affected by this inhibitor. As shown in Fig. 5h Ned-19 treatment down-regulates VEGFR2 expression starting from 24 h suggesting a further mechanism through which NAADP inhibition exerts its antitumor effects.

## Discussion

VEGF/VEGFR2 complex represents a promising target for therapeutic strategies aimed at inhibiting neoangiogenesis. We have recently identified a novel VEGFR2-specific signaling pathway that crucially controls *in vivo* neoangiogenesis through NAADP-operated release of Ca^2+^ from intracellular acidic stores^33^. This previous finding prompted us to investigate whether endogenously-induced angiogenesis taking place in a solid tumor could be similarly impaired by pharmacological inhibition of NAADP signal. The evidence we obtained and report here goes beyond expectation, showing that in a murine model of highly aggressive and highly metastatic tumor, known to secrete VEGF^35^,^40^, Ned-19 inhibits neoangiogenesis, formation of metastases and tumor growth, unexpectedly exerting its action also on tumor cells. The conditions in which angiogenesis occurs in a normal tissue site following administration of a single growth factor differ in many ways from those in which it takes place, spontaneously, in the context of a solid tumor. First of all a variety of cell types are present at the tumor site, contributing to the local microenvironment^41^. The specific and complex interplay of local signals characterizing tumor microenvironment is known to originate from a number of variables such as the heterogeneity of tumor cell population and the extent of stromal cell involvement^42^,^43^. Moreover, differences between normal and tumor vessels, not only in morphology and in functionality but also in origin and mode of formation are well known^44^,^45^,^46^. As a consequence of this dynamic complexity the specific microenvironment in which a pharmacological inhibitor is tested is far from defined, and the cascade of events linking the intended initial target to the final outcome is not easily identified. The experiments performed on melanoma cell cultures were in fact aimed at providing some clues in this direction and the data obtained have allowed for some deductions on the *in vivo* counterpart. Below, data from the two experimental approaches are analyzed and discussed in parallel.

Pharmacological treatment with NAADP inhibitor is usually performed *in vitro*. To our knowledge, *in vivo* use of Ned-19 has been reported so far only from our laboratory^33^ as a component of Matrigel plugs implanted in mice for angiogenesis experiments. In the present research, the systemic effects of repeated i.p. administration of Ned-19 were preliminary tested on weight, diastolic pressure, and pulse rate. These parameters were chosen as it has been shown^47^ that rats administered with NAADP-AM (the cell permeant NAADP derivative) undergo decrease in arterial pressure. In our experiments, the first to our knowledge testing the drug in animals, Ned-19 did not substantially affect either pulse rate or weight, while diastolic pressure remained within physiological values; in fact, after a minor 24 h raise, it appeared stabilized within 48 h after treatment. Considering these parameters and pharmacokinetic data, every second day administration of 5 mg/Kg was chosen as a safe protocol to investigate the effect of Ned-19 on angiogenesis and tumor growth in mice inoculated with B16 cells.

The antitumor effect of NAADP inhibition on B16-derived tumors was apparent and manifold. I.p. Ned-19 administration in B16 tumors bearing mice significantly impaired tumor growth compared to vehicle treated specimens. Moreover, the density of CD31 positive tumor vessels as well as the total number of lung metastases were strongly reduced by Ned-19 treatment.

More than one scenario could be envisaged when attempting to interpret these data. In the most reductive one, a linear chain might link all of the effects: Ned-19 would abolish the angiogenic response of ECs to locally produced VEGF^48^,^49^, in line with what known to occur in HUVEC^33^. Impaired development of vascularization would then result in poor support of tumor growth and inhibition of metastatic dissemination. A more likely hypothesis envisages further contributions to this basic cascade converging to the same end point through a more complex interplay.

The possibility that tumor cells themselves could be sensitive to the inhibitor was taken into consideration in cell culture experiments. Treatment of B16 cells with Ned-19 was found to reduce the cell migratory ability paralleled by reduction in FAK activation, down-regulation of N-cadherin and up-regulation of E-cadherin expression. In accordance with this data it is known that intra-and extravasation of tumor cells, fundamental for metastatic invasion, involve the expression of adhesion molecules such as cadherins and FAK, mediating the contact with endothelial and subendothelial surfaces^50^. In the development of cutaneous melanoma, decreased expression of E-cadherin and increased expression of N-cadherin accompany cell detachment and transendothelial migration^51^ which is a critical process in cancer development and metastasis. Moreover, Ned-19 was found to reduce cell proliferation and time-dependently induce cell apoptosis. Interestingly, we observed that NAADP-inhibition induced a remarkable down regulation of VEGFR2 expression in B16 melanoma cells. The whole of these findings strongly indicate that these tumor cells are sensitive to the inhibition of NAADP-mediated Ca^2+^ signaling. A NAADP-operated system seems in fact to be active in the tumor cells themselves, controlling functions as relevant as proliferation, VEGFR2 expression and migratory abililty. Experiments of calcium imaging showed that in response to 100 μg/L VEGF, B16 cells undergo an [Ca^2+^]_i_ release which is controlled by NAADP dependent acidic stores. The observed inhibitory effects of Ned-19 in unstimulated B16 cultures is therefore likely to indicate that these cells respond to autocrinally secreted VEGF by means of a NAADP-controlled Ca^2+^ signaling pathway. In consideration of the well known presence of VEGF in melanoma microenvironment^35^ it seems possible that this signaling pathway is active also *in vivo*, hence to hypothesise that it has contributed to the overall inhibitory effect of Ned-19 treatment in our *in vivo* model. The observed direct effect of Ned-19 on tumor cell viability and growth most likely entails altered production of tumor-derived proangiogenic factors. In as far as is legitimate to transfer *in vitro* evidence to *in vivo* models, the impaired vascularization of tumors in Ned-19 treated mice would then result not only from direct inhibition of EC response to VEGF^33^ but also from reduced supply of angiogenic factors. This framework is no doubt compatible with the possibility that *in vivo* Ned-19 may have affected additional targets, possibly interfering at multiple levels with the complex intercellular crosstalk specific of the tumor microenvironment.

Whatever the detailed roles of further possible players, the detection of a new level of vulnerability in the aggressive behaviour of melanoma represents a step forward towards the identification of potential therapeutic targets. Moreover, to our knowledge this is the first report on NAADP system involvement in tumor cells, a finding of broader potential interest, given the possibility that this Ca^2+^-mobilizing second messenger might play a crucial role in the biology of additional types of tumor cells.

## Material and Methods

### Chemicals and reagents

The following antibodies were used: Rabbit anti-phospho-FAK (Tyr925, Cell Signaling Technologies, Danvers, MA, USA), purified Mouse anti-N-cadherin (BD Biosciences, New Jersey, USA), Mouse anti-E-cadherin (Invitrogen, Life Technologies, Monza, Italy), Mouse anti-VEGFR2 (R&D, Minneapolis, MN), β-Actin HRP-conjugated (Sigma, Milano, Italy), stabilized Goat anti-Mouse HRP-conjugated (Thermo Fisher Scientific, Waltham, MA), stabilized peroxidase conjugated Goat anti-Rabbit [(H + L), Thermo Fisher Scientific]. Reagents used are: VEGF-A165 (Peprotech, Rocky Hill, NJ, USA), trans-Ned-19 (Tocris Bioscences, Bristol, United Kingdom), Bafilomycin A1 (Sigma), Thapsigargin (Sigma). Ned-19 was purchased from Tocris Bioscience and was at least 98% pure. Methanol and other organic solvents were chromatography grade from Sigma, water was purified using a milliQ system from Millipore (Billerica, MA, USA) and LC-MS grade formic acid was obtained from Fluka (Sigma). Centrifugal ultrafiltration devices were Amicon Ultra-0.5 (Ultracel-3 K, regenerated cellulose 3,000 MW) from Millipore Co. Ltd.

### Pharmacokinetic analysis

Mice were administered a single i.p. dose of 5 mg/Kg Ned-19. At times from 0 min to 24 h after administration individual mice were sacrificed, blood samples were taken by cardiac puncture, centrifuged at 250 g for 10 min at 4 °C and the resulting plasma were stored at −80 °C until analyzed. 75 μL mouse plasma was mixed with 75 μL methanol. The mixture was vortexed for 1 min and applied to a 500 μL centrifugal filter. The capped tube was then centrifuged at 12,000 g for 15 min at 25 °C. Eight microliter of the ultrafiltrate solution was directly injected into the HPLC system and Ned-19 analysis was performed as follow by liquid chromatography tandem mass spectrometry (LC-MS/MS). The HPLC equipment consists of a Series 200 Micro-LC Pump and a Series 200 autosampler from Perkin Elmer (Norwalk, CT, USA). A triple quadrupole mass spectrometer, AB-Sciex API 2,000 (Toronto, ON, Canada) was used for detection. The analyte was analysed using a C_18_ phase Kinetex column (10 cm × 2.1 mm ID) from Phenomenex (Torrance, CA, USA) packed with 2.6 μm core–shell particles. A Phenomenex security GuardUltra Cartridge was also used to protect the column from damaging contaminants and microparticulates. The mobile phases were (A) methanol and (B) water, both containing 5% formic acid, at a flow rate of 0.4 mL/min and were entirely transferred into the mass spectrometer source. The gradient elution was as follows: increase of the organic phase from 0 to 5% in 1 min, then to 30% in the following minute and linearly to 100% in 0.5 min. Finally, after 1 min of 100% methanol the organic phase was reduced to the original 0% in 2.5 min to enable equilibration of the column. The resulting total run time was 6 min. Ned-19 was detected in positive ionization with a capillary voltage of 5,500 V, nebulizer gas (air) at 45 psi, turbo gas (nitrogen) at 80 psi and 425 °C. The other ion source parameters were set as follows: curtain gas (CUR), 25 psi; collision gas (CAD), 5 psi; declustering potential (DP), 60 V; entrance potential (EP), 12 V. Instrument conditions optimization was performed by direct infusion and manual tuning. A 100 ng/mL methanolic solution was prepared for this purpose. Data collection and elaboration were performed by means of Analyst 1.4 software (AB- Sciex). The quantitative data were acquired using Multi Reaction Monitoring (MRM) mode. Two MRM transitions (precursor ion > fragment ion) were selected for the analyte, namely *m*/*z* 515.2 > 442.1 and 515.2 > 262.2. Collision energy (CE) was set at 23 and 38 eV while collision cell exit potential (CXP) was at 22 and 13 V for the two transitions respectively.

Ned-19 stock solutions were prepared by dissolving analyte in the appropriate amount of methanol in order to obtain individual stock solutions at 1,000 μg/mL. The stock solution was further diluted to obtain standard stock solutions containing 100 and 1 μg/mL analyte. All the stock solutions were stored at −20 °C. The analytical method was validated according to FDA guidelines for bioanalytical method validation. Linearity, recovery, matrix effect, precision, accuracy, limits of detection (LODs) and lower limits of quantification (LLOQs) were evaluated. Calibration standard solutions were prepared in water: methanol (50/50, v/v) by dilution of the appropriate stock solution; the calibration range was 1 to 500 ng/mL; the calibrators were prepared at 7 concentrations. Precision, recovery and accuracy were evaluated at 3 concentrations (1, 75, 500 ng/mL) and the resulting values were found to fall within acceptable limits. The extraction recovery for Ned-19 was 69 ± 6%. Matrix effect was not significant over the linearity range (<10%). The limit of detection (LOD) was defined as the lowest concentration with a signal-to-noise (S/N) ratio greater than 3. The limit of quantification (LOQ) was defined as the concentration at which both precision (RSD %) and accuracy were less than 20%. To this purpose mouse plasma spiked with the analyte and subjected to the extraction step at concentrations ranging from 0.2 to 2 ng/mL were analysed in triplicate. LLOQ was found to be 0.5 ng/mL while LOD was 0.2 ng/mL.The validated method was then successfully applied in measuring Ned-19 following drug injection in mice to support the pharmacokinetic study.

### B16 melanoma cell line culture

B16 murine melanoma cell line provided by Dr. L. Morasca (Mario Negri Institute, Milan, Italy) was maintained in Dulbecco’s modified Eagle’s medium (Sigma) supplemented with 2 mmol/L glutamine (Sigma), 10% fetal calf serum (Sigma), and antibiotics (P/S, Sigma).

### B16 tumor cells transplant in syngenic mice

Six to ten-week-old male C57BL/6 mice were purchased from Charles River Laboratories (Calco, LC,Italy). The animals were housed at the University of Rome Histology Unit accredited animal facility, in individual cages in an environmentally controlled room (23 °C, 12 h light–dark cycle) and provided with food and water ad libitum. All of the procedures were approved by the Italian Ministry for Health and conducted according to the US National Institutes of Health guidelines. 4 × 10^6^ B16 cells were resuspended in 200 μl PBS. The cells suspension was subcutaneously injected into the flank of the mice. Tumors were grown to ≈50–100 mm^3^ before starting treatment with Ned-19 or vehicle (DMSO). I.p. injection of 5 mg/Kg Ned-19 was performed every 2 days for 4 weeks. Tumor volume based on caliper measurements was calculated by the modified ellipsoidal formula (tumor volume = ½ × (length × width)^2^).

### Histology and immunohistochemistry

Formalin-fixed lungs and zinc-fixed tumors were dehydrated and embedded in paraffin, serial 4-μm-thick paraffin sections were stained with Haematoxylin-Eosin (H&E) or used for immunohistochemistry^52^. The number of lung macro metastases was macroscopically evaluated while micro metastases were microscopically studied at 20× magnification, as reported^53^. The number of tumor vessels per mm^2^ was evaluated at 200× magnification on zinc-fixed, rat monoclonal anti-CD31 stained sections (BD Pharmingen, San Diego, CA, USA), using amino-9-ethylcarbazole (AEC) as chromogen, Haematoxylin counterstaining and positive and negative controls^53^. Double-blind measurements were performed in at least 10 randomly selected fields for each specimen with the exclusion of necrotic areas, with an interobserver variability less than 5%.

### Calcium imaging

B16 cells cultured on 35-mm dishes were incubated in culture medium containing 3.5 μmol/L FURA-2-AM (Invitrogen) for 1 h at 37 °C, and then rinsed with Hanks’ Balanced Salt Solution (HBSS, Sigma). Each dish was placed into a culture chamber at 37 °C on the stage of an inverted fluorescence microscope (TE2000E, Nikon Instruments, Fi, Italy), connected to a cooled CCD camera (512B Cascade, Roper Scientific, Ottobrunn, Germany). Samples were illuminated alternately at 340 and 380 nm using a random access monochromator (Photon Technology International, New Jersey, USA) and emission was detected using a 510 nm emission filter. Images were acquired (1 ratio image per s) using Metafluor software (Universal Imaging Corporation, Downington PA, USA). Calibration was obtained at the end of each experiment by maximally increasing intracellular Ca^2+^-dependent FURA-2AM fluorescence with 5 μmol/L ionomycin (ionomycin calcium salt from Streptomyces conglobatus, Sigma) followed by recording minimal fluorescence in a Ca^2+^-free medium. [Ca^2+^]_i_ was calculated according to the formulas previously described^54^.

### Boyden chamber assay

The cell ability to migrate was evaluated by the Boyden chamber assay, which makes use of a chamber composed of two medium-filled compartments separated by a microporous membrane (8 μm pore size, BD Bioscences). The lower well contained medium with 10% (vol/vol) serum. Cells were placed in the upper chamber in complete medium containing either Ned-19 or vehicle and allowed to migrate through the pores of the membrane into the lower compartment. After 24 h the membrane was fixed and Hoechst stained, and the number of cells that had migrated to the lower side of the membrane was evaluated in a Zeiss Axioscope microscope (Zeiss, Mi, Italy). The number of migrating cells from three independent experiments were counted in 10 random view-fields per well and the values averaged.

### Western Blot

Western blot was performed on B16 cells treated with Ned-19 for 24, 48 and 72 h. The intensity of bands was quantified by Image J software (NIH) from at least three independent experiments, normalized to β-actin content and compared with vehicle (DMSO)-treated controls (set as 1).

### MTT test

To perform the methylthiazolyldiphenyl-tetrazolium (MTT, Sigma) test as a measurement of cell proliferation 5 mg/mL MTT was dissolved in PBS and filtered. B16 cells were cultured, detached using trypsin/EDTA, then added onto 96-well plates at a concentration of 10 × 10^4^ cells in 200 μL DMEM per well and incubated in the specific experimental condition for 24 h. As a control for background absorption, cells were omitted in some of the wells. After incubation the medium was removed, 100 μL serum-free medium (DMEM) containing 0.5 mg/mL MTT solution was added to each well and the plate was incubated in a humidified 5% (vol/vol) CO_2_ incubator at 37 °C for 3 h to allow MTT to be metabolized. Then, 100 μL DMSO (Sigma) were added to each well, pipetting up and down to dissolve crystals, and optical density was read at 570 nm.

### Flow cytometric assessment of cell viability and growth

Cells were treated with Ned-19 or DMSO (vehicle). After 24, 48, 72 h, cells were harvested by trypsin/EDTA (Sigma), rinsed with PBS + 1% BSA (Sigma) and incubated with 1 μg/mL propidium iodide (PI, Sigma). Cells were then analysed using a Beckam Coulter Epics XL flow cytometer (Beckman Coulter, Fullerton, CA) and data were analysed using FCS Express5 Flow Research Edition software (De Novo Software, Glendale, CA) to determine the number of dead vs alive cells for each experimental condition.

### Cell cycle analysis

Propidium iodide (PI) staining: cells were detached with trypsin/EDTA, washed once with PBS, resuspended in 50% FCS in PBS and then fixed in 70% ethanol for 24 h. After washing three times with PBS, cells were incubated with 50 μg/mL PI plus 0.1 U/L RNAse for 3 h at room temperature before FACS analysis by Coulter Epics XL flow cytometer (Beckman Coulter). Cells were gated using both area vs peak to eliminate doublets and forward vs side scatter to exclude debris. The percentage of cells in the different cycle phases and in subG1 (composed of dead cells and debris) was calculated using FCS Express5 Flow Research Edition software.

### Annexin V-FITC staining

Cells were harvested by trypsin digestion, which was blocked by addition of serum followed by a wash in PBS, then treated with binding buffer containing PI and/or annexin V-FITC (Immunological Sciences, Rome, Italy) following the manufacturer’s instructions, prior to FACS analysis. Annexin V-FITC positive cells, either PI-negative or low PI positive, were considered as apoptotic. Since Ned-19 has been reported to fluoresce^31^, a procedure was preliminarily devised to circumvent any possible interference with annexin V-FITC fluorescence. To this aim we set up a gate on cells unstained with annexin V-FITC and found that the median of fluorescence intensity (MFI) was 2.5 times higher in Ned-19- treated versus untreated cells. Therefore the FL1 positivity marker for Ned-19 treated samples was set to include the Ned-19 treated cells not stained with annexin V-FITC in the lower left quadrant.

### Statistical analysis

Data are presented as the mean ± s.e.m. of results from at least three independent experiments. Student t test was used for statistical comparison between means where applicable. *P < 0.05; **P < 0.01; ***P < 0.0001.

## Additional Information

**How to cite this article**: Favia, A. *et al.* NAADP-Dependent Ca^2+^ Signaling Controls Melanoma Progression, Metastatic Dissemination and Neoangiogenesis. *Sci. Rep.* **6**, 18925; doi: 10.1038/srep18925 (2016).
